# On Whether Task Experience of the Peer Differentially Impacts Feedback Scheduling and Skill Acquisition of a Learner

**DOI:** 10.3389/fpsyg.2019.01987

**Published:** 2019-09-10

**Authors:** Jae Todd Patterson, Matthew McRae, Steve Hansen

**Affiliations:** ^1^Department of Kinesiology, Brock University, St. Catharines, ON, Canada; ^2^Faculty of Education & Professional Studies, School of Physical and Health Education, Nipissing University, North Bay, ON, Canada

**Keywords:** motor learning, knowledge of results, feedback, self-control, practice schedule

## Abstract

Previous research has shown that peers without task experience provided knowledge of results (KR) as effectively as performers who self-controlled their own KR schedule (McRae et al., 2015). In the present experiment, a group of participants first practiced a motor task while self-controlling their KR during a defined acquisition period. Twenty-four hours after their last retention trial, these participants with motor experience then provided KR to a learner during their skill acquisition. Participants were required to learn a serial-timing task with a goal of 2,500 ms. Participants completed a defined acquisition period and then returned 24 h later for a retention test. In retention, learners who received KR from experienced peers were predicted to outperform learners who received KR from inexperienced peers. The results showed that performers learned the task similarly, independent of the peer’s previous task experience. However, the peer groups differed in their frequency of providing KR to the learner and showed a discrepancy between their self-reported KR provision strategy and when they actually provided KR. The results have theoretical implications for understanding the impact of self-control in motor learning contexts.

## Introduction

Reducing a performer’s uncertainty regarding the success of a goal-directed motor action is facilitated by the provision of augmented feedback. Augmented feedback is information about a performance or the outcome of an action provided by an external source such as a coach or instructor. The augmented feedback schedule experienced by the learner can either facilitate or undermine skill acquisition. For example, feedback provided too frequently, too immediately, or without control of the learner has shown to undermine skill acquisition of the learner (Salmoni et al., 1984; Wulf et al., 2010). In other examples, feedback presented less frequently or on a schedule that is under the control of the learner have shown to facilitate skill acquisition (Wulf et al., 2010; Wulf and Lewthwaite, 2016). Augmented feedback in the form of knowledge of results (KR) is a source of information provided to a learner that is related to the outcome of their movement action with reference to the movement goal (Salmoni et al., 1984). Recently, our understanding of the informational role of KR has been extended by examining KR schedules that are controlled by the learner. This paradigm has been termed a self-controlled KR schedule. The results from research affording the learner control over their receipt of KR suggests that learners are utilizing the information from the KR display to (1) confirm a “good” trial rather than a “poor” trial based on a frequently reported preference for KR after perceived good trials (Chiviacowsky and Wulf, 2002, 2005; Wulf, 2007; Chiviacowsky et al., 2008; Patterson and Carter, 2010; Hansen et al., 2011; Chiviacowsky et al., 2012; Patterson et al., 2013; Kaefer et al., 2014; c.f., Patterson et al., 2011; Aiken et al., 2012; Laughlin et al., 2015) and (2) strengthen their error detection capabilities as a result of actively processing task-related intrinsic feedback before requesting KR (Carter and Patterson, 2012).

Findings from research examining self-controlled KR schedules have provided insight into understanding the mechanisms underlying the learning advantages. Two alternative perspectives have been proposed. First, from an information processing perspective, providing the learner with control over their KR schedule is believed to enhance the meaningfulness and informational value of KR such that KR is only requested when based on a self-reported preference, such as a perceived good trial. Specifically, with the act of requesting or declining feedback, learners are predicted to actively engage in interpreting their task-related intrinsic feedback in order to formulate an estimate regarding the success of their just-completed trial and then make a decision whether or not they prefer to receive KR (Chiviacowsky and Wulf, 2002, 2005; Chiviacowsky et al., 2012). The additional active cognitive engagement in deciding upon whether feedback is necessary is purported to assist learning. In support of this notion, recent research has shown that blocking the ability of the learner to process their task-related intrinsic feedback in the pre-KR interval with a cognitive dual task undermined the learning advantages of a self-controlled KR context (Carter and Ste-Marie, 2017). Similarly, if learners are required to decide whether or not they prefer to receive KR before completing the motor action, the learning advantages of a self-controlled KR practice are similarly undermined (Carter et al., 2014). This information processing perspective highlights the importance of facilitating the cognitive engagement in interpreting task-related intrinsic feedback in the post-response pre-KR period before deciding upon the receipt of KR. Second, an alternative perspective suggests that providing the learner with the opportunity to self-control their KR schedule is supporting the learner’s basic psychological need to be in a learning context that is autonomy-supportive context (Ryan and Deci, 2000; Sanli et al., 2013; Wulf and Lewthwaite, 2016). Providing the learner autonomy within a practice context is believed to not only fulfill a psychological need but also subsequently enhance the motivation to learn the task and increase self-efficacy (see Wulf and Lewthwaite, 2016 for review). Although these two theoretical perspectives offer viable explanations for the mechanisms underlying the learning advantages of a self-controlled KR context, they cannot account for the learning similarities between a learner-controlled KR context and a KR schedule controlled by a peer, as shown in McRae et al. (2015).

The results from McRae et al. (2015) showed that learners who received KR from a peer without task experience and those performers self-controlling their KR demonstrated similar learning. In addition to the motor performance similarities between the two conditions, McRae et al. (2015) also reported that participants in the self-controlled KR condition preferred requesting KR after perceived good trials, consistent with previous self-controlled KR research (e.g., Chiviacowsky and Wulf, 2005; Bastos et al., 2018). Peers without task experience preferred to provide KR to the learner after both good and bad trials equally. Yet, learners reported they were satisfied with the KR schedule organized by the peer, despite the fact that they received feedback after good and bad trials equally, but reported that they would prefer feedback after good performance trials. Karlinsky and Hodges (2014) also showed similar learning advantages for performers self-controlling the repetition schedule during multi-task learning and a repetition schedule that was organized by a peer see also (Karlinsky and Hodges, 2018).

The findings from the peer-controlled research are rather curious since the learners are not afforded control, but are showing similar learning to those learners who are provided control. Further, the peers who are providing feedback have not had previous experience with the task. Currently, it is unknown whether a peer with task experience would supersede the learning advantages of a peer without task experience. Findings from educational research offer some insight. Results from peer-teaching research have shown that fellow peers were inferior in providing useful feedback to a learner compared to that of an expert (i.e., teacher) based on a web-based project of the same student (e.g., Hovardas et al., 2014). Further, mixed dyads consisting of an experienced and inexperienced peer have shown superior learning compared to two inexperienced peers (Asterhan et al., 2014). In other instances, the educator was considered to have a primary role in the effectiveness of peer-assisted learning, such that the progression from educator-facilitated learning to peer-assisted learning was determined by the educator (Sevenhuysen et al., 2015). Currently, it is unclear in the motor learning literature whether the experience of the peer and their subsequent KR schedule would differentially impact skill acquisition of a learner.

To address this gap in knowledge, the purpose of the present experiment was to examine whether task experience of the peer would differentially impact the KR schedule of the peer and subsequent motor skill acquisition of the learner. The peers in McRae et al. (2015) had no previous experience with the motor task or experience with individualizing a KR schedule for the to-be-learned motor task. Specifically, we were interested in whether a peer’s previous experience with the motor task would differentially modulate (1) the frequency of providing KR to a performer, (2) the peer’s preference for providing KR to a performer (e.g., good trials, poor trials, both, etc.), and (3) subsequent skill acquisition of the participant receiving KR from a peer with or without task experience.

Firstly, we predicted that learners paired with an experienced or inexperienced peer would demonstrate similar motor performance during the acquisition period (e.g., Karlinsky and Hodges, 2014; McRae et al., 2015). This prediction was based on research that has identified that when KR is made available to a learner, a learner can experience informational and motivational benefits (for review, see Wulf et al., 2010). Furthermore, it has been identified that when KR is available during acquisition, KR can equate the motor performance of experimental conditions (Salmoni et al., 1984; Wulf and Shea, 2002; Kantak and Winstein, 2012). Secondly, for the delayed retention test, we predicted that learners who received KR from peers with task experience would demonstrate superior motor performance compared to learners who received KR from inexperienced peers (Hovardas et al., 2014). This is especially so, since previous research has shown that the learning advantages of a self-controlled KR practice context are undermined when requests to receive KR are decided before completing the motor trial (Chiviacowsky and Wulf, 2005; Carter et al., 2014). These findings highlighted the meaningfulness of task-related intrinsic feedback in the post-response pre-KR period when deciding whether to receive KR of the just completed trial. We believed that this opportunity afforded to the experienced peer would prove advantageous in controlling another peer’s KR schedule. Thirdly, based on previous self-reports, peers without previous task experience were expected to prefer to provide KR after relatively good and bad trials equally (McRae et al., 2015) whereas the peers with task experience were expected to provide KR more frequently after relatively poor trials (Bjerrum et al., 2014). This prediction was based on results from the peer-teaching research showing that inexperienced peers were inferior in improving another learner’s performance compared to an experienced peer (Hovardas et al., 2014). Finally, we predicted that peers (i.e., inexperienced and experienced peers) would provide their paired learners with more frequent KR over the course of the acquisition phase than the frequency of KR requested by the learners in the self-control condition (e.g., McRae et al., 2015).

## Materials and Methods

### Participants

Sixty individuals were recruited from the university student body (20 men and 40 women; *M* = 21.9 ± 1.8 years). All participants were self-declared right handed and wore corrective lenses when prescribed such that everyone had normal vision during testing. Participants received course credit upon completion of the experiment. Written informed consent was acquired. This study received ethical approval from the University Research Ethics Board under the protocol number #14-004.

### Apparatus

A custom-made E-Prime Software program (E-prime version 2.0.8.74 Psychology Software Tools, Inc., Pittsburgh, PA, United States) controlled the timing of the experimental stimuli and recorded the timing and accuracy data. The software was run on a Dell OptiPlex computer with an Intel^®^ Core^TM^ i5-2500 CPU @3.30 GHz processor. Experimental stimuli were presented on two 19″ flat-screen Dell monitors with display settings set to 1,290 × 1,024 pixels with a refresh rate of 60 Hz. Manual responses were recorded using a Psychnet Tools five-key serial response box (Psychological Software Tools, Inc., Pittsburgh, PA, United States). All responses were made through the depression of four keys on the response box. Visual stimuli were presented in black, 12-point, Arial font with a white background. Liquid crystal goggles (Translucent Technologies Inc., Toronto, ON, Canada) were used to occlude the learners’ vision during various time points of the experimental protocol. The “learner” also wore a pair of industrial headphones to avoid verbal interaction with the peer.

### Task

Participants were asked to complete a computerized timing task (see Hansen et al., 2011). The task required participants to respond to a series of numbers (3-1-2-4-3-1) appearing on a computer monitor by depressing the corresponding buttons on a five-key serial response box. The buttons on the serial response box were numbered from left (#1) to right (#5) and participants were asked to depress the buttons with their index finger of their non-dominant hand. The goal of the motor task was to complete the sequence in exactly 2,500 ms.

### Procedure

Participants were pseudo-randomly assigned to the experienced peer condition (EP, *n* = 12), the learner with an “experienced peer” condition (L-EP, *n* = 12), the “inexperienced peer” condition (IP, *n* = 12), the learner with an “inexperienced peer” condition (L-IP, *n* = 12), or the control condition (CO, *n* = 12). Similar to McRae et al. (2015), participants in the inexperienced peer (IP) condition had no previous experience with the motor task prior to providing KR to a performer. Participants assigned to the EP condition completed the protocol while self-controlling their KR schedule during the acquisition period and then they returned approximately 24 h after their last retention trial to provide KR to a participant in the L-EP condition during their acquisition period. The CO group observed the EP group member’s KR displayed on the CO participant’s computer screen when the EP participant requested KR during the acquisition period. When KR was not requested by the EP participant, the CO participant viewed a blank computer screen for 5 s, similar to the EP participant. The purpose of the CO group was to control for potential social influence that the paired participants might experience during the acquisition period. All pairings consisted of learners of the same sex (i.e., Male–Male; Female–Female). Participants assigned to the role of a peer controlling the KR schedule of a learner were informed that their role was to provide feedback to optimize learning (i.e., retention) of the motor skill. Based on the experimental setup, the peer and the learner did not communicate during the experimental protocol. The learners were unaware of the experience level of the peer during the acquisition period.

### Acquisition

Two standardized computer desks were positioned facing in opposite directions in such a way that the participants sat back to back, approximately 2 m from each other. The desk facing the left side of the lab was labeled “Desk 1” and the desk facing the right side of the lab was labeled “Desk 2.” Desk 1 was occupied by the “peer” (i.e., IP and EP participants) and the other participants (i.e., LI, LE, and CO groups) were seated at Desk 2.

Each acquisition trial started with the word “Ready?” presented in the middle of the computer screen for 3,000 ms. A sequence of six numbers (3-1-2-4-3-1) was then displayed in serial order. Upon completion of each response, including any incorrect button pushes, the next number in the sequence (i.e., “1”) appeared on the screen until six responses were recorded. Total response time was recorded from the first button push to the final button push of the six-key sequence. Upon depression of the final button in the sequence, “Trial Complete” was presented for 500 ms in the center of the computer screen.

Participants in the EP condition were queried whether or not they wanted KR regarding their just completed acquisition trial: “Would you like feedback? Y/N?” If they chose “yes,” they depressed the “Y” button on the keyboard and then were immediately provided the following feedback display: (1) Task Goal: 2,500 ms; (2) whether the sequence was completed correctly or incorrectly; (3) Too fast/Too slow; and (4) Constant error (ms). This feedback was presented to the learner for 5,000 ms followed by a “done” screen for 1,000 ms. If the participant decided not to receive KR on a just completed trial, they depressed the “N” key on the keyboard and a “done” screen was presented for 6,000 ms. During the acquisition period, a participant of the control condition sat at Desk 1 and viewed the KR, when requested by the EP participant, on a separate computer monitor. Participants in the EP condition were informed that the participant in the control condition was observing the outcome of their performance on a separate computer screen. Participants in the EP condition returned approximately 24 h later for a no-KR retention test. Twenty-four hours after the last delayed retention trial, participants in the EP condition returned to the laboratory for a third time as an experienced peer who then determined when to provide KR to a participant in the L-EP condition.

Participants assigned to receive KR from a peer with (L-EP condition) or without (L-IP condition) previous task experience would view a “Trial Complete” screen for 500 ms upon completion of every acquisition trial. Their vision was then occluded by the liquid crystal goggles for the duration of time required by the peer to determine whether or not to provide KR to the performer. During this time, the peer viewed the KR from the performer’s just completed acquisition trial. The KR provided to the peer was identical to the KR provided to participants in the EP condition. Peer facilitators were then asked: “Would you like to provide feedback Y/N?” If they chose “yes,” the peer depressed the “Y” button. Conversely, if the peer wished to withhold feedback, then they depressed the “N” button. As soon as the peer entered their response, the goggles worn by the learner became transparent. On KR trials, the learner viewed their KR from the just completed trial for 5,000 ms followed by a done screen for 1,000 ms. On no-KR trials, the word “done” was presented for 6,000 ms (see Table 1).

**TABLE 1:** Experimental protocol for experimental conditions during the pre-test, acquisition, delayed retention and transfer test (EP, experienced peer; IP, inexperienced peer; L-EP, learner with inexperienced peer; L-EP, learner with experienced peer; CO, control condition).

**Day**		**Experimental condition**					
		
			**Experienced peer (EP)**		**Control (CO)**
1		Pre-test	10 no-KR practice trials with task		–
		Acquisition	80 practice trials; self-controlled KR		Observe EP perform motor task for 80 trials
2		Delayed retention		30 no-KR practice trials	

		**Experienced peer (EP)**	**Inexperienced peer (IP)**	**Learner with inexperienced peer (L-IP)**	**Learner with experienced- peer (L-EP)**

3	Pre-test	X	X	10 no-KR practice trials with task	
	Acquisition	Self-control KR for L-EP participant for 80 trials	Self-control KR for L-IP participant for 80 trials	KR schedule controlled by IP for 80 acquisition trials	KR provision controlled by EP for 80 acquisition trials
	Delayed retention	X	30 no-KR trials		

Following the completion of the acquisition period, all participants completed a short paper-and-pencil questionnaire regarding their feedback schedule during the acquisition period (e.g., Chiviacowsky and Wulf, 2002). Participants in the EP condition were asked the following: (1) When/why did you ask for feedback? (2) When did you not ask for feedback? For each question, participants were required to choose from the following options: Perceived good trial, perceived poor trial, perceived good and poor trials equally, randomly, or other (see McRae et al., 2015).

Participants who assumed the role of a peer were asked the following: (1) When/why did you provide feedback? (2) When did you not provide feedback to the learner? Options for both questions included the following: perceived good trial, perceived bad trial, perceived good and bad trials equally, randomly, or other. The peers were also queried in regard to how effective they thought their KR schedule was with the following: “How much do you believe your feedback schedule was successful at improving the learning of your paired participant?” The peer participant circled a value ranging from 1 (“Ineffective”) to 10 (“Extremely Effective”).

The learners in the L-IP and L-EP groups, who physically practiced the task and were provided KR from a peer, were asked to respond to the following: (1) Do you think you received feedback after the right trials? (Yes/No). (2) If NO, when would you have liked to receive feedback? Options for question 2 included: perceived good trial, perceived bad trial, perceived good and bad equally, randomly, or other. Additionally, these participants were asked: “How much do you believe the feedback schedule provided to you was successful at improving your learning of the task?” Participants were asked to circle a number between 1 (“Ineffective”) and 10 (“Extremely Effective”).

Approximately 24 h after the final acquisition trial, participants in the L-EP and L-IP conditions returned to complete 10 no-KR trials of the acquisition task. The total duration of the delayed retention test was approximately 15 min.

### Dependent Measures and Analyses

During the acquisition phase, the proportion of KR requests and KR provisions (i.e., EP, IP) during the acquisition period were analyzed in a 2-Group (IP and EP) by 8-Block ANOVA with repeated measures on blocks. The performance of participants physically practicing the task (i.e., L-IP, L-EP, and EP groups) was indexed using measures of absolute error (AE), constant error (CE), variable error (VE), and the number of errors committed. These measures were analyzed in four separate 3-Group (L-IP, L-EP, and EP) by 8-Block mixed ANOVAs with repeated measures on the last factor. AE from KR and no-KR trials during acquisition was analyzed in a 3-Group (L-IP, L-EP, and EP groups) by 2-Feedback Choice (KR and no-KR) ANOVA with repeated measures on Feedback Choice.

Motor performance in the retention test was indexed by AE, CE, and VE. The measures were analyzed in separate 4-group (L-IP, EP, L-EP, and CO) ANOVA. Tukey’s HSD *post hoc* tests were used during follow-up analyses involving more than two means. The statistical significance level for this study was set at *p* < 0.05. Effect sizes were reported as partial eta squared ($\eta_{p}^{2}$). Feedback preference questionnaire data were presented as descriptive statistics. We corrected for violations of sphericity by using the Greenhouse-Geisser procedures where required.

## Results

### Acquisition

#### Proportion of KR Trials

The 2-Group (IP, EP) by 8-Block (1–8) ANOVA with repeated measures on block was performed to assess whether the frequency of feedback to the learner differed as a function of task experience of the peer. Analysis of feedback frequencies for the two peer conditions showed a main effect for Group, *F* (1, 22) = 7.32, *p* = 0.013, $\eta_{p}^{2}$ = 0.25. Peers with task experience provided KR less frequently to the learners compared to inexperienced peers (see Table 2).

**TABLE 2:** Acquisition mean scores (SD) for the proportion of feedback trials provided by the inexperienced peer (L-IP) and the experienced peer group (L-EP).

**Groups**	**Block 1**	**Block 2**	**Block 3**	**Block 4**	**Block 5**	**Block 6**	**Block 7**	**Block 8**
L-IP	0.68 (0.23)	0.61 (0.21)	0.65 (0.26)	0.58 (0.25)	0.51 (0.25)	0.58 (0.29)	0.63 (0.23)	0.60 (0.31)
EP	0.58 (0.29)	0.50 (0.34)	0.55 (0.33)	0.41 (0.28)	0.48 (0.32)	0.51 (0.31)	0.49 (0.21)	0.68 (0.22)
L-EP	0.58 (0.25)	0.45 (0.24)	0.47 (0.22)	0.33 (0.20)	0.33 (0.18)	0.30 (0.13)	0.34 (0.17)	0.36 (0.22)

*Additionally, the proportion of feedback trials requested by learners with self-control (EP).*

#### AE on Trials With KR Compared to No-KR Trials

Analyses revealed a significant interaction of Group × KR trial, *F* (2, 32) = 5.68, *p* < 0.01, $\eta_{p}^{2}$ = 0.26. The L-IP were provided KR on trials with greater AE (*M* = 227.2, *SD* = 107.1) compared to lower AE on no-KR trials (*M* = 142.7, *SD* = 72.7). AE on trials of L-EP participants was not statistically different on KR (*M* = 199.2, *SD* = 86.3) and no-KR (*M* = 182.2, *SD* = 122.5). These results suggest that the IP peers preferred to provide KR after attempts that could be considered “bad” attempts, whereas the EP peers did not demonstrate a preference for providing KR as a function of AE. The EP during their task acquisition period also did not show a preference for KR as a function of AE based on KR (*M* = 207, *SD* = 79.6) and no-KR-trials (*M* = 243.7, *SD* = 83.9).

#### Feedback Preference Questionnaire

Self-report data showed that 8 out of the 12 performers (67%) with an inexperienced peer (i.e., L-IP group) believed that they received feedback after their preferred trials during the acquisition period. In comparison, 11 out of 12 performers (92%) with experienced peers (i.e., L-EP group) reported that they received feedback after their preferred trials. Participants who received feedback from the inexperienced peer rated the effectiveness of their KR schedule [ranging from 1 (ineffective) to 10 (extremely effective)] as 7.3/10. Participants receiving feedback from the experienced peer reported the effectiveness of their feedback schedule to be 7.7/10 (see Table 3). Inexperienced and experienced peers rated the perceived effectiveness of their KR respective schedule for facilitating skill acquisition of their performer [from 1 (ineffective) to 10 (extremely effective)] as 6.8/10 and 6.9/10, respectively. In summary, the peer facilitators rated the perceived effectiveness of the KR schedule that they created lower than the learners who received that KR schedule.

**TABLE 3:** Feedback questions for learners: the effectiveness of feedback scale that was used ranged from 1:10 with verbal anchors of 1 (Ineffective), 5 (Moderately Effective), and 10 (Extremely Effective).

**Number of responses**	
**Learner with an inexperienced peer group (L-IP)**	
1. Do you think you received feedback after the right trials?	
(a) Yes	8
(b) No	4
2. If NO, when would you have liked to receive feedback?	
(a) Perceived good trials	0
(b) Perceived bad trials	2
(c) Perceived good and bad equally	0
(d) Randomly	0
(e) Other	2
3. How well did the feedback schedule provided to you facilitate your learning of this task?	7.3/10
**Self-control to peer experienced facilitator group (EP)**	
As learner with self-control	
1. When/Why did you request feedback?	
(a) Perceived good trials	4
(b) Perceived bad trials	1
(c) Perceived good and bad equally	3
(d) Randomly	1
(e) Other	3
2. When/Why did you not request feedback?	
(a) Perceived good trials	1
(b) Perceived bad trials	5
(c) Perceived good and bad equally	1
(d) Randomly	3
(e) Other	2
**Learner with an experienced peer group (L-EP)**	
1. Do you think you received feedback after the right trials?	
(a) Yes	11
(b) No	1
2. If NO, when would you have liked to receive feedback?	
(a) Perceived good trials	1
(b) Perceived bad trials	0
(c) Perceived good and bad equally	0
(d) Randomly	0
(e) Other	0
3. How well did the feedback schedule provided to you facilitate your learning of this task?	7.7/10

#### Absolute Error

There was a main effect of Block, *F* (3.49, 115.24) = 13.65, *p* < 0.01, $\eta_{p}^{2}$ = 0.29. Block 1 (*M* = 318.1, *SD* = 185.7) had greater AE compared to block 2 (*M* = 215.0, *SD* = 101.6), block 3 (*M* = 203.2, *SD* = 103.6), block 4 (*M* = 159.5, *SD* = 84.7), block 5 (*M* = 150.9, *SD* = 77.9), block 6 (*M* = 171.4, *SD* = 100.5), block 7 (*M* = 187.0, *SD* = 127.2), and block 8 (*M* = 161.1, *SD* = 82.2). Block 2 (*M* = 215.0, *SD* = 101.6) and block 3 (*M* = 203.2, *SD* = 103.6) demonstrated higher AE than block 5 (*M* = 150.9, *SD* = 77.9; see Table 4).

**TABLE 4:** Acquisition and retention mean scores (standard deviations) for absolute error (AE), constant error (CE), and variable error (VE) (ms) as a function of block and experimental condition (L-IP, learner with inexperienced peer; L-EP, learner with experienced peer; EP, participants controlling their own KR schedule; CON, control condition).

**Group**	**Block 1**	**Block 2**	**Block 3**	**Block 4**	**Block 5**	**Block 6**	**Block 7**	**Block 8**	**Retention**
**AE (ms)**									
L-IP	261 (105)	189 (98)	201 (88)	164 (94)	156 (75)	172 (88)	179 (118)	156 (88)	374 (303)
EP	364 (168)	212 (55)	240 (129)	164 (95)	170 (98)	153 (81)	244 (169)	165 (81)	238 (61)
L-EP	330 (254)	244 (136)	169 (83)	151 (71)	128 (56)	189 (131)	138 (53)	162 (85)	187 (119)
CON									746 (570)
**CE (ms)**									
L-IP	−148(178)	−87(67)	−59(150)	−9(83)	−39(101)	−26(139)	−51(137)	−31(105)	−9(325)
EP	28 (372)	−59(125)	22 (201)	11 (141)	−5(131)	−10(100)	83 (239)	16 (108)	−7(204)
L-EP	42 (363)	−52(242)	−83(59)	−16(116)	−25(80)	−25(219)	−51(81)	−64(152)	−64(199)
CON									677 (626.8)
**VE (ms)**									
L-IP	195 (76)	140 (74)	156 (83)	120 (74)	103 (48)	135 (85)	137 (121)	117 (101)	171 (93)
EP	228 (104)	141 (34)	255 (202)	145 (103)	120 (86)	108 (47)	284 (44)	118 (71)	156 (80)
L-EP	177 (81)	117 (38)	114 (40)	143 (157)	83 (25)	125 (77)	84 (22)	105 (62)	110 (37)
CON									298 (124)

#### Constant Error

The Group × Block interaction was not statistically significant, *F* (4.99, 82.44) = 0.88, *p* = 0.50, $\eta_{p}^{2}$ = 0.05. The main effects were also non-significant [Group, *F* (2, 33) = 1.88, *p* = 0.17, $\eta_{p}^{2}$ = 0.10; Block, *F* (2.49, 82.44) = 0.53, *p* = 0.81, $\eta_{p}^{2}$ = 0.02 (see Table 4)].

#### Variable Error

The analyses revealed a main effect of Block, *F* (7, 231) = 2.96, *p* = 0.005, $\eta_{p}^{2}$ = 0.08. Block 1 (*M* = 200.2, *SD* = 87.9) had higher VE compared to block 2 (*M* = 132.9, *SD* = 51.5), block 4 (*M* = 136.2, *SD* = 113.7), block 5 (*M* = 102.1, *SD* = 58.9), block 6 (*M* = 122.5, *SD* = 70.4), and block 8 (*M* = 113.2, *SD* = 77.7). Furthermore, block 3 (*M* = 174.9, *SD* = 138.1) had higher VE compared to block 5 (*M* = 102.2, *SD* = 58.9; see Table 4).

#### Number of Errors Committed

The Group × Block interaction was not statistically significant, *F* (14, 231) = 0.87, *p* = 0.60, nor was the group main effect, *F* (2, 33) = 0.06, *p* = 0.94, or the block main effect, *F* (7, 231) = 0.99, *p* = 0.44.

### Delayed Retention Test (No-KR Test 24 H After Acquisition)

#### Absolute Error

The analyses revealed a main effect of Group, *F* (3, 44) = 7.03, *p* < 0.01, $\eta_{p}^{2}$ = 0.32. The CO group (*M* = 745.6, *SD* = 569.8) performed the task with higher AE compared to the LE group (*M* = 187.3, *SD* = 119.1), the SCP group (*M* = 237.6, *SD* = 61.1), and the LI group (*M* = 294.9, *SD* = 135.4). All other between-group differences were not statistically significant.

#### Constant Error

There was a main effect of Group, *F* (3, 44) = 7.19, *p* < 0.01, $\eta_{p}^{2}$ = 0.34. Similar to the AE analyses, the *post hoc* test showed that the CO group (*M* = 676.6, *SD* = 626.8) performed the task with greater CE compared to the LE group (*M* = −64.3, *SD* = 198.6), SCP group (*M* = −6.7, *SD* = 204.8), and LI group (*M* = −8.4, *SD* = 325.3; see Table 4).

#### Variable Error

Analyses of VE revealed a main effect of Group, *F* (3, 44) = 9.73, *p* < 0.01, $\eta_{p}^{2}$ = 0.40. The CO group (*M* = 297.9, *SD* = 124.4) performed the task with greater VE compared to the LE group (*M* = 110.5, *SD* = 36.9), SCP group (*M* = 155.4, *SD* = 79.9), and LI group (*M* = 170.8, *SD* = 92.6; see Table 4).

#### Number of Errors Committed

There were no statistically significant differences between groups for the average number of errors committed during the retention test, *F* (4, 55) = 1.06, *p* = 0.38.

## Discussion

The purpose of the present experiment was to determine whether the previous motor task experience of the peer would differentially impact the effectiveness of the peer’s KR scheduling and the subsequent skill acquisition of a learner. McRae et al. (2015) showed that a KR schedule organized by a peer without previous task experience was as effective for skill acquisition compared to participants who self-controlled their own KR. The present experiment extends the work of McRae et al. (2015) by including a group of participants who acquired task experience before determining the KR schedule of another participant during acquisition. We were specifically interested in whether a peer’s previous experience would modulate (1) the frequency of KR provision, (2) the peer’s preference strategy for providing KR, and (3) subsequent skill acquisition of the participant receiving KR from a peer. We predicted that learners paired with an experienced or inexperienced peer would demonstrate similar motor performance during the acquisition period (e.g., Karlinsky and Hodges, 2014; McRae et al., 2015). This prediction was supported. For the retention period however, we predicted that learners who received KR from a peer with experience would demonstrate superior motor performance in the retention period compared to learners who received KR from inexperienced peers. This prediction was based on the established importance of experiencing task-related intrinsic sensory feedback (Wolpert et al., 2011) before deciding whether to receive KR (Carter et al., 2014) during motor skill learning. Further, Carter et al. (2014) showed that learning was superior for participants experiencing task-related intrinsic feedback before requesting KR compared to those participants requesting KR at the beginning of a trial, before experiencing task-related feedback (see also Chiviacowsky and Wulf, 2005). This prediction was also based on results from the peer-teaching research showing that inexperienced peers were inferior in improving another learner’s performance compared to an experienced peer (e.g., Hovardas et al., 2014).

We also predicted that peers, independent of previous task experience, provide their paired learners with more frequent KR over the course of the acquisition phase than the frequency of KR requested by the learners in the self-control condition (e.g., McRae et al., 2015). This prediction was also not supported since the peers with experience provided KR less frequently than the inexperienced peers during the acquisition period.

Further, based on task experience of the peer, we predicted that inexperienced peers would provide KR more commonly after relatively good and bad trials equally (e.g., McRae et al., 2015) whereas the experienced peers would commonly provide KR after relatively poor trials (e.g., Bjerrum et al., 2014). Independent of previous task experience, peers reported a preference for providing KR after relatively poor trials. A discussion of our results follows.

To our knowledge, this was the first experiment to show that previous task experience of the peer did not differentially impact skill acquisition of a learner compared to a peer providing feedback without previous task experience. This finding extends McRae et al. (2015) who showed that receiving feedback from a peer without previous task experience was as effective for skill acquisition as participants self-controlling their feedback during the acquisition period. Our findings are also concomitant with Karlinsky and Hodges (2014) who showed that inexperienced peers scheduled the repetition order of multiple motor tasks as effectively as a learner self-controlling their repetition order. Recall that we predicted that peers with previous task experience who were afforded the opportunity to experience task-related sensory information before requesting their own KR were expected to organize an optimal KR schedule for the learner. Other findings in the self-controlled KR research have shown that the opportunity to request KR has proven more advantageous after a motor action, compared to making the decision before a motor action (Chiviacowsky and Wulf, 2005; Carter et al., 2014). As a result, the request for KR has been predicted to be more meaningful after experiencing task-related sensory information (Carter et al., 2014). However, our findings did not support this prediction. Previous task experience did not differentially impact motor performance of the learner during the delayed retention test. Our findings also do not support findings in the education literature showing that when learning in pairs (i.e., dyads), an inexperienced peer partnered with an experienced peer was more advantageous to learning for the inexperienced peer, compared to two inexperienced peers partnered together (e.g., Asterhan et al., 2014; Bjerrum et al., 2014). Inexperienced peers are suggested to lack the ability to appropriately identify or correct errors in another learner’s performance (e.g., Hovardas et al., 2014). However, Cho and MacArthur (2010) suggests that peers without experience can more easily relate to the learner of the same skill level, such that feedback is provided that is consistent with the preferences of the learner. Future research is required to further examine the impact of the amount of previous task experience of the peer and the impact of this experience of KR scheduling for a learner.

Similar to McRae et al. (2015), the results of the present experiment challenge the theoretical importance of preserving autonomy of the learner during motor skill learning. The learning advantages of a self-controlled KR context have been explained via two theoretical perspectives. From an information processing perspective, performers required to self-control their KR schedule are believed to engage in greater processing of task-related information such that meaningfulness of the KR is strengthened (Carter et al., 2014) and the error detection capabilities of the learner are also strengthened (Carter and Patterson, 2012). From an autonomy-supportive perspective, practice contexts designed to support a learner’s autonomy are suggested to enhance motivation of the participant and subsequent skill acquisition (Wulf and Lewthwaite, 2016). Curiously, McRae et al. (2015) showed that learners provided KR from a peer without task experience showed similar learning to peers provided the opportunity to control the receipt of KR. Further, Karlinsky and Hodges (2014) also showed that the learning of multiple motor tasks was similar between participants afforded the opportunity to control their practice schedule and those who had their practice schedule organized by a peer without task experience. Since, in both experiments, the performer without autonomy over their practice context learned the task as effectively as those afforded autonomy, we suggest that providing the peer the responsibility of scheduling KR for the learner enhanced their motivation to ensure optimal learning of their participant, independent of their previous task experience. Evidence for the leaning advantages associated with providing a peer, rather than the learner, autonomy over the feedback schedule produces a unique extension to the research examining self-controlled KR schedules. The learning advantages of practice contexts under control of the learner have been attributed to the learning advantages of practice contexts that are autonomy-supportive (see Wulf and Lewthwaite, 2016). Autonomy-supported practice environments, compared to those that are not, are suggested to optimize a learner’s motivation and self-efficacy and then to subsequently enhance the performer’s expectancy for success and motor skill acquisition (see Wulf and Lewthwaite, 2016 for review). Our results, as well as the findings from others (e.g., Karlinsky and Hodges, 2014; McRae et al., 2015), suggest that learning in a peer-controlled context can be similar to a learner-controlled context. Recent research may offer some insight into our findings. Daou et al. (2016) showed that participants who were told they would be required to teach the motor skill they were currently learning showed superior learning in the retention period compared to those groups of participants not provided this information. Thus, providing the peer control over the learner’s KR schedule perhaps enhanced the peer’s motivation to maximize learning of the performer they were controlling KR for. This is perhaps especially so since peers were instructed to provide feedback in a manner that enhanced the performance and learning of the participant. In fact, participants self-reported being quite satisfied with their KR schedule being controlled by the peer.

In our current experiment, the experienced peers, who practiced the motor task before providing feedback to a learner, requested feedback on 52.2% of their own acquisition trials, but provided feedback to learners on 39.6% of the acquisition trials. Peers without task experience provided KR on 60.3% of the acquisition trials. Since participants in both peer conditions were providing KR to participants learning a motor task, the feedback frequencies were expected to be similar between the peer groups and similar to other experiments with peers providing KR to a learner (McRae et al., 2015). This prediction was not supported. Peers with task experience provided KR less frequently compared to the peers without task experience. The higher frequency of KR trials provided by peers without task experience was similar to the frequency of KR provided by the peers without task experience in McRae et al. (2015) (*M* = 0.64). Despite the statistical difference in feedback frequencies, retention performance was not statistically different between learners receiving feedback from an experienced or inexperienced peer. Based on the fact that learners were not receiving KR on all trials during the acquisition period, independent of the peers’ previous task experience, and motor performance was similar in the retention period between peer conditions, we suggest that a reliance on KR to guide motor performance was not evidenced as a function of the KR schedule experienced (Salmoni et al., 1984).

Recall that we predicted that peers without previous task experience would provide KR after both relatively good and bad trials equally (McRae et al., 2015), whereas the peers with task experience would provide KR after relatively poor trials (Bjerrum et al., 2014). This prediction was only partially supported. Most peers with task experience self-reported a preference for providing KR after perceived poor trials (33% of peers) as did most peers without task experience (50% of peers). The self-reported findings from the peers without task experience did not support our prediction and are contrary to the findings of McRae et al. (2015) who showed that the peers preferred to provide feedback after relatively good and poor trials equally. Curiously, the preference self-reported by the peers in the present experiment is also contrary to the preference self-reported by learners in past self-controlled feedback conditions. Previous self-controlled KR research has shown a preference for learners to request feedback more frequently after perceived good, rather than perceived poor trials (Chiviacowsky and Wulf, 2002, 2005; Wulf, 2007; Patterson and Carter, 2010; Hansen et al., 2011; c.f., Aiken et al., 2012; Laughlin et al., 2015; Bastos et al., 2018). This preference has been attributed to the decreased cognitive processing demands attributed to repeating the motor commands of a correct response, compared to engaging in the cognitive demands required to fix an error (Lam et al., 2010). However, the peers in the present experiment seemingly preferred a feedback schedule that was consistent with placing heightened cognitive demands on the learner (e.g., correct an error versus repeating a successful motor plan).

To determine whether this was in fact the case, we examined AE on KR vs. no-KR trials to determine whether the preferred and actual feedback schedule was commensurate. For example, if AE was higher on KR compared to no-KR trials, the peers preferred strategy (i.e., poor trials) was commensurate with their actual strategy. The results showed that the peers without task experience were consistent with their preferred (i.e., poor trials) and actual KR trials (i.e., higher AE on KR trials). This finding is consistent with McRae et al. (2015) who also showed that the preferred and actual KR trials were consistent for the peers without task experience. However, the peers with task experience did not show a consistency between their reported preferred (i.e., after relatively poor trials) and actual selection (e.g., good and poor trials) based on AE on KR and no-KR trials. Further research is required to understand the strategies underlying the peers’ KR schedule as a function of task experience.

From the learner’s perspective who was receiving KR from a peer, we were interested in (1) whether the learner was satisfied with the feedback schedule organized by their peer, and (2) whether the peer perceived that their feedback schedule was effective in facilitating the skill acquisition of their learner. In the present experiment, 67% of learners receiving feedback from a peer without task experience and 92% of learners with an experienced peer self-reported they received feedback on the trials they would have also preferred to receive feedback. These findings support those of McRae et al. (2015) who also showed that performers were satisfied with the feedback schedule received by their peer without task experience. Participants receiving feedback from a peer were also asked to rate the perceived effectiveness of their feedback schedule received from the peer for facilitating their skill acquisition. The learners receiving KR from an experienced peer (*M* = 7.7) and inexperienced (*M* = 7.3) peer facilitators rated the perceived effectiveness of their feedback schedule similarly. When the peers were asked about the perceived effectiveness of their feedback schedules, the peers without (*M* = 6.8) and with task experience (*M* = 6.9) also rated the effectiveness of their feedback similar. Of interest, participants in the present experiment were unaware of the experience level of their peer providing feedback during the acquisition period. Further research is required to determine whether being cognizant of the experience of the peer would differentially impact the perception of the effectiveness of the skill level and subsequent learning of the motor skill.

## Conclusion

In summary, the findings from the present study extend our understanding of the utility of peer-controlled KR schedules by including peers with previous task experience. Previous research by McRae et al. (2015) showed that peers without previous task experience individualized a KR schedule for performers that was as beneficial for learning as performers provided the opportunity to control their own KR schedule. We extend upon those results by showing that previous task experience of the peer failed to differentially impact the motor skill retention of the learner. However, previous task experience of the peer modulated the characteristics of the KR schedule. For example, peers without previous task experience provided KR more frequently compared to peers with task experience. However, the learners receiving KR from a peer self-reported satisfaction in regard to when KR was provided and in its utility in facilitating acquisition of the motor task. Additional research is recommended to further examine whether knowing the status of the peer facilitator (experienced vs. inexperienced) would differentially impact skill acquisition of the learner and their perceived effectiveness of the KR schedule on their skill acquisition. Participant being cognizant of a skill difference between themselves and their peer may either undermine or facilitate the perceived effectiveness of the KR schedule. In summary, there are numerous skill acquisition contexts whereby performers receive feedback from other performers, who may either be similar, such as a classroom peer or teammate, or dissimilar, such as a coach or teacher from their current skill level. Our results suggest that peers differing in motor task experience can provide KR in a manner that does not undermine learning of their fellow peer.

## Ethics Statement

This study was carried out in accordance with the recommendations of the Research Ethics Board of Brock University with written informed consent from all subjects. All subjects gave written informed consent in accordance with the Declaration of Helsinki. The protocol was approved by the Social Sciences Research Ethics Board of Brock University.

## Author Contributions

All authors listed have made a substantial, direct and intellectual contribution to the work, and approved it for publication.

## Conflict of Interest Statement

The authors declare that the research was conducted in the absence of any commercial or financial relationships that could be construed as a potential conflict of interest.
